# Influence of Delivery Strategy on Message-Processing Mechanisms and Future Adherence to a Dutch Computer-Tailored Smoking Cessation Intervention

**DOI:** 10.2196/jmir.2153

**Published:** 2013-02-06

**Authors:** Nicola Esther Stanczyk, Rik Crutzen, Catherine Bolman, Jean Muris, Hein de Vries

**Affiliations:** ^1^Maastricht University/CAPHRIDepartment of Health PromotionMaastricht UniversityMaastrichtNetherlands; ^2^Open University of the NetherlandsDepartment of PsychologyOpen University of the NetherlandsHeerlenNetherlands; ^3^Maastricht University/CAPHRIDepartment of General PracticeMaastrichtNetherlands

**Keywords:** computer tailoring, smoking cessation, message-processing mechanisms, e-loyalty, future adherence

## Abstract

**Background:**

Smoking tobacco is one of the most preventable causes of illness and death. Web-based tailored smoking cessation interventions have shown to be effective. Although these interventions have the potential to reach a large number of smokers, they often face high attrition rates, especially among lower educated smokers. A possible reason for the high attrition rates in the latter group is that computer-tailored smoking cessation interventions may not be attractive enough as they are mainly text-based. Video-based messages might be more effective in attracting attention and stimulating comprehension in people with a lower educational level and could therefore reduce attrition rates.

**Objective:**

The objective of the present study was to investigate whether differences exist in message-processing mechanisms (attention, comprehension, self-reference, appreciation, processing) and future adherence (intention to visit/use the website again, recommend the website to others), according to delivery strategy (video or text based messages) and educational level, to a Dutch computer-tailored smoking cessation program.

**Methods:**

Smokers who were motivated to quit within the following 6 months and who were aged over 16 were included in the program. Participants were randomly assigned to one of two conditions (video/text CT). The sample was stratified into 2 categories: lower and higher educated participants. In total, 139 participants completed the first session of the web-based tailored intervention and were subsequently asked to fill out a questionnaire assessing message-processing mechanisms and future adherence. ANOVAs and regression analyses were conducted to investigate the differences in message-processing mechanisms and future adherence with regard to delivery strategy and education.

**Results:**

No interaction effects were found between delivery strategy (video vs text) and educational level on message-processing mechanisms and future adherence. Delivery strategy had no effect on future adherence and processing mechanisms. However, in both groups results indicated that lower educated participants showed higher attention (*F*_1,138_=3.97; *P*=.05) and processing levels (*F*_1,138_=4.58; *P*=.04). Results revealed also that lower educated participants were more inclined to visit the computer-tailored intervention website again (*F*_1,138_=4.43; *P*=.04).

**Conclusions:**

Computer-tailored programs have the potential to positively influence lower educated groups as they might be more involved in the computer-tailored intervention than higher educated smokers. Longitudinal studies with a larger sample are needed to gain more insight into the role of delivery strategy in tailored information and to investigate whether the intention to visit the intervention website again results in the ultimate goal of behavior change.

**Trial Registration:**

Netherlands Trial Register (NTR3102).

## Introduction

During the last decade, an increasing number of people used the Internet to obtain health-related information. In the field of health promotion, the Internet has become an important medium for the delivery of behavioral change interventions [1,2]. Health professionals have started to deliver several lifestyle behavior interventions through the web, including interventions aimed at smoking cessation [3], nutrition behavior [4], and physical activity [5,6].

A successful interactive strategy consists of computer-tailored interventions [7] through which individuals receive personalized information and feedback on health behavior and motives for this behavior. Tailored health messages are based on a person’s answers to a questionnaire on individual characteristics related to health behavior. Current research has shown the superiority of tailored materials over existing standard materials [8,9]. In the field of smoking cessation, recent studies have revealed computer-tailored smoking cessation interventions to be more effective than non-tailored interventions [9,10]. Yet, as with many eHealth interventions, smoking cessation interventions delivered via the Internet have high attrition rates, especially among people with lower education [11,12]. However, less educated people are often those who smoke more cigarettes [13] and show higher nicotine addiction rates, less quit-attempts, and more relapses compared to people with a higher level of education [14,15]. Past research in the Netherlands has shown that smoking prevalence was significantly higher among less educated people (29%) compared to people with a higher level of education (20%) [13]. Smokers of lower socioeconomic status (LSES) are therefore a highly relevant target group for using these computer-tailored programs. Hence, it is important to investigate how attrition rates among lower educated groups can be reduced and how computer-tailored interventions can be optimized to attract groups of different education levels.

A possible reason for the high attrition rates in computer-tailored interventions is that they rely heavily on text-based messages. Research suggests that video-based messages might be more effective in attracting attention and stimulating comprehension in people with a lower educational level [16,17]. Furthermore, video-based messages have been shown to require less mental effort and may help the person focus on the core elements of the message [18,19]. It is therefore conceivable that video-based messages may be better for reaching lower educated groups and realizing behavior change. Additionally, higher educated groups may benefit more from in-depth processing and accordingly may be stimulated more by text-based messages [20].

Recent research examining computer tailoring identified different underlying message processing-mechanisms that play an important role in enhancing health communication [21]. In tailored communication, five important message-processing mechanisms have been identified: attention, comprehension, processing, self-reference, and appreciation. Attention refers to the ability to focus on the receiving message. Due to the fact that paying less attention to a message lowers the overall effect, one purpose of tailored material is to increase the attention to the message. A recent study has shown stronger attention processes in people reading tailored material [22]. Comprehension refers to the ability to understand the content of the message. Past studies have shown that the better the message was matched to personal attributes and preferences, the more the message was understood and remembered [23,24]. A psychological theory that has been used to explain the effects of tailoring is the Elaboration Likelihood Model (ELM). This theory states there are two processing routes, the peripheral and the central. Related to this theory, personally relevant messages are processed by the “central route” and therefore take more effort to process [25]. The idea of tailoring is to increase the perceived relevance of the message in order to elicit a careful consideration of the message, which leads to a deeper impact of the received content. Indeed, a study about weight loss information indicated participants engaged in deeper processing of tailored information when compared to non-tailored information [26]. Besides effortful processing, a further aim of tailoring is to stimulate self-referential thinking. Self-referential thinking refers to the ability to refer the given information to one’s own situation. For example, tailored weight loss materials have been shown to encourage participants to link the information to their own situation [26]. Furthermore, it is also theorized that tailoring influences the appraisal of a message. Tailored materials may enhance the feeling of being well understood and would therefore lead to more appreciation compared to non-tailored information. To sum up, tailoring is used to increase the relevance of a health message by stimulating attention, comprehension, and the overall depth of message processing. The effects of tailoring on message-processing mechanisms might be further increased by the use of a suitable delivery strategy. However, as already indicated, these possible effects might be different for people with a lower or higher educational level.

To our knowledge, no previous studies have empirically examined the effects of delivery strategies (video vs text) and their impact on message-processing mechanisms among different educational groups. The first aim of this paper was to explore whether there exist differences in message-processing mechanisms according to delivery strategy (video or text based messages) and educational level. Past research has shown that information processing in lower educated groups was less profound and more influenced by visual than textual information [27]. Based on this, it was expected that tailoring would result in deeper information processing in lower educated smokers who received the video-based messages. In contrast, it was expected that the effects of tailoring would increase for higher educated smokers who received the text-based messages.

Additionally, eHealth research has acknowledged the importance of user experience of the intervention website. User experience refers to what a person thinks and feels during and after being exposed to a website [28]. Past research demonstrated the importance of user experiences (eg, trustworthiness, enjoyment) with regard to revisiting the website [29]. A positive user experience is related to an increased website use [28], resulting in future adherence [30]. Future adherence can comprise two components: (1) the intention to revisit an Internet-delivered intervention, and (2) recommending an Internet-delivered intervention to others [30]. Internet-delivered interventions and especially computer-tailored interventions often consist of several modules/feedback sessions. Since several sessions are often necessary to achieve behavior change in the long run [31,32], it is important to know whether people would like to revisit the intervention website. People with a high intention to revisit the website intervention might be likely to follow further important parts/sessions of the intervention and could therefore benefit more from the intervention than people leaving the program after their first visit. Next, it is important to know whether people would recommend the Internet-delivered intervention to others since previous studies demonstrated that the “word of mouth” strategy is effective in order to increase the use of eHealth interventions [33]. In order for web-based interventions to have an impact on public health, it is important that the intervention is also disseminated by the target population [34]. Now that computer-tailored interventions have become an increasingly popular strategy in the field of smoking cessation interventions, it is important to examine whether future adherence of computer-tailored intervention websites is influenced by delivery strategy (video vs text) and whether these effects vary among different educational groups.

The second aim of this study was to investigate whether there exist differences in future adherence according to delivery strategy (video or text based messages) and educational level. It was expected that future adherence would increase in lower educated smokers who received the video-based messages; whereas, we expected the same effects for higher educated smokers who received the text-based messages.

In summary, the current study was designed to assess whether a different delivery strategy (video vs text) interacted with educational level on message-processing mechanisms and future adherence of a Dutch computer-tailored smoking cessation program.

## Methods

### Background

This study aims to provide an in-depth exploration of the effects of video and text computer tailoring on message-processing mechanisms, which are also tested within the currently tested RCT [35]. Hence, the sample and the study we describe in this paper are different from the currently tested RCT. The described study uses only one session of the intervention tested within the RCT to test a different hypothesis (ie, differences between video/text on outcome measures regarding message-processing mechanisms and future adherence, which are not directly related to health, and not the hypothesis stated in the RCT protocol concerning the effect on smoking cessation) and was therefore not registered as a trial.

### Sample

Participants for this study were recruited in May 2011 through the Dutch Internet research agency Flycatcher [36]. From this panel, a sample of potential participants (N=11,583) was approached to fill in a short web-based questionnaire about their smoking behavior, their motivation to quit, and their educational level. The main purpose of this pre-analysis was to include participants who smoked only at the time of the study inclusion, were aged 16 years or older, were motivated to quit, and could be categorized as lower or higher educated participants. They were first asked to indicate whether they smoked. Participants who indicated they smoke were next asked to indicate how often they smoked ranging from daily to once a month or less. Motivation to quit smoking was measured by one item assessing whether the participant intended to quit smoking in the future on an 8-item scale ranging from “definitely not” (1) to “definitely yes” (8). All participants who at least indicated that they were most likely (6) to quit in the near future were categorized as motivated. Educational level was divided into low (primary, basic vocational, lower general school), intermediate (higher general secondary education, preparatory academic education, medium vocational school) and high (higher vocational school or university level). Only participants with a low or high educational level (categorized to the standards of Statistics Netherlands) were invited to take part in the study [37]. In total, a random sample of panel members (N=300) who met these inclusion criteria was invited to participate in the study.

**Figure 1:**
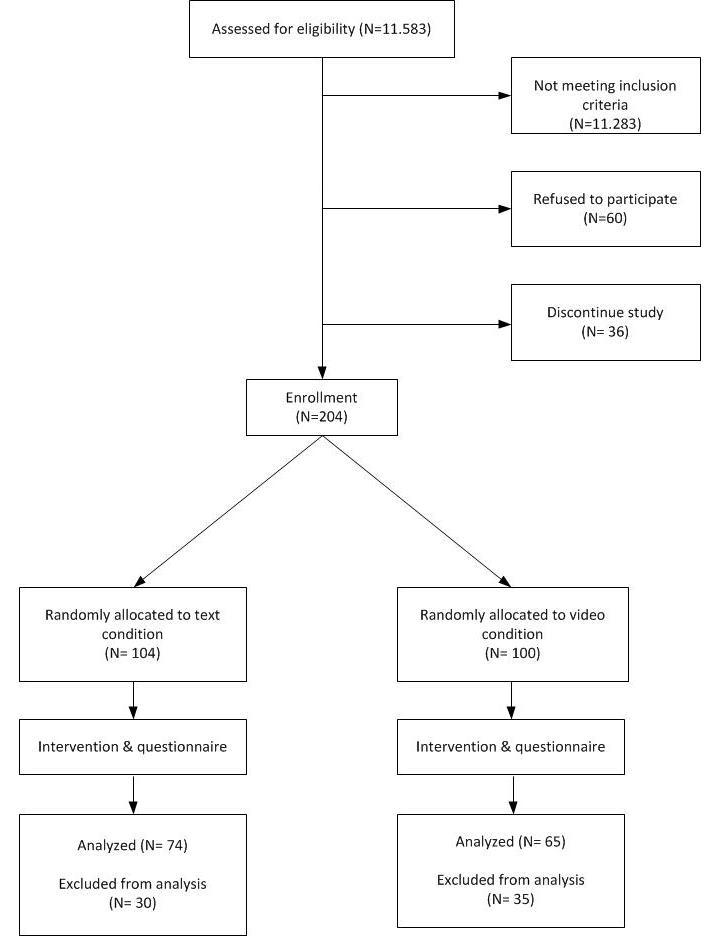
Flowchart of the study.

From this sample, 240 clicked on the link of the intervention website and agreed to participate in the study (response rate: 80%); 36 participants did not fill out the questionnaire resulting in a sample of 204 participants who finished the study (retention rate: 85%). From these 204, only smokers who stayed more than 5 minutes on the intervention website (n=139) were included in the main analysis. We used this inclusion criterion since a minimum of 5 minutes is needed to process the information in both conditions. In order test our hypotheses on message-processing mechanisms and future adherence correctly, we found it necessary to include only smokers who thoroughly completed the program. This resulted in a sample of 139 participants (see Figure 1). As an additional strategy, we also conducted an intention-to-treat analysis of the data from all participants, also including those who stayed less than 5 minutes on the website.

### Procedure

After signing up for participation and giving online informed consent, all included smokers were exposed to the website of a Dutch smoking cessation computer-tailored intervention [38]. Participants were informed that they were able to discontinue their participation in the study at any time without any consequences. Smokers were randomly allocated to either the text condition or the video condition. Allocation to the two conditions was executed by the Tailor Builder computer software program (OSE, Sittard, the Netherlands). This software was developed for the execution of different web-based tailored interventions [39]. Smokers had to follow only one session of the currently tested computer-tailored smoking cessation intervention. After completing this session, they were asked to fill out a web-based questionnaire assessing message-processing mechanisms and the intention to revisit the website and recommend it to others.

### Content

#### Intervention

**Figure 2:**
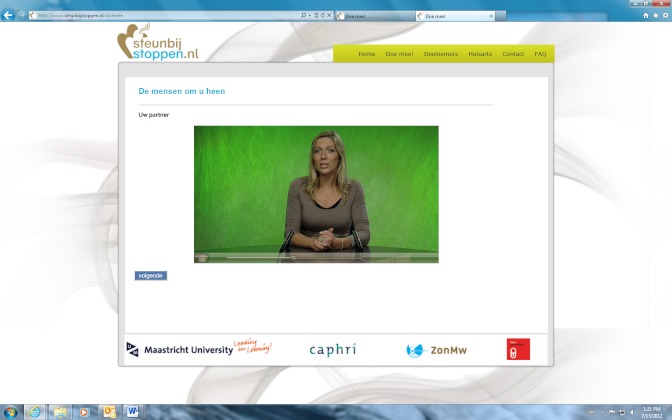
Screenshot of personal advice from the video condition.

**Figure 3:**
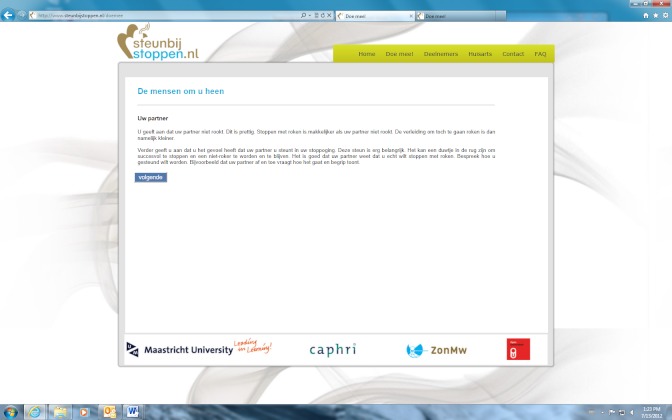
Screenshot of personal advice from the text condition.

The computer-tailored smoking cessation intervention was based on the I-Change model [40]. Participants in the text condition received computer-tailored text messages, whereas participants in the video condition received computer-tailored video messages. In the text condition, participants were presented text phrases without any further graphics or animations. For the video condition, the text-driven messages were translated into narrated video-driven messages that had a news-driven format with different adults delivering the tailored messages. We used simple videos without any other animation effects such as cartoons, hyperlinks, etc. In the video condition, the same tailored advice was used as in the text condition. The only difference between the two conditions was the strategy of delivery; the content of the advice was the same in both conditions (see Figures 2 and 3) Feedback messages were based on a participant’s answers to a questionnaire and tailored to their individual characteristics, such as their beliefs towards smoking, their intention to quit, and their overall smoking behavior. The session that smokers had to follow in this study was intended to increase participants’ motivation to quit smoking and to encourage smokers to quit smoking in the near future. First, smokers received three tailored feedback messages on their perceived advantages and disadvantages of quitting. Next, one piece of advice was offered with respect to participants’ perceived social support. Last, one piece of tailored advice was provided on their perceived self-efficacy to quit smoking. A detailed description of the different intervention components that are assessed in the currently tested RCT are reported elsewhere [35].

#### Measures

The following demographic variables were measured: age, gender (0=male; 1=female), and educational level (0=low; 1=high) [37]. Furthermore, time spent on the intervention website was logged by the program system in minutes and seconds.

*Addiction level* was measured by 6 items using the Fagerström Test for Nicotine Dependence (FTND), asking participants how many cigarettes they smoked per day, at which time points, and whether they had difficulties not smoking in smoke-free places. The answers were converted into an overall sum score (0=not addicted; 10=highly addicted) [41].

*Readiness to quit smoking* was measured by one item asking participants whether they intended to quit smoking, resulting in 6 categories (6=yes, within the following month; 5=yes, within 1-3 months; 4=yes, within 4-6 months; 3=yes, within 1 year; 2=yes, within 1-5 years; 1=yes, but not within the following 5 years) [42,43].

*Cognitive processing* (eg, “I like tasks where I do not have to think much”, assessing to what extent people engage in effortful processing) was measured by 6 items on a 5-point scale of the Heuristic Systematic Processing Questionnaire ranging from 5, “I totally agree” to 1, “I totally disagree” (Cronbach alpha=.74) [44].

Future adherence was measured by two concepts: intention and recommendation. *Intention to revisit* (eg, “It is likely that I will visit the website again in the future”) was measured by 3 items on a 7-point scale, ranging from 7, “I totally agree” to 1, “I totally disagree” (Cronbach alpha=.91). *Recommendation* to others (eg, “It is likely that I will recommend this website to others”) was measured by 3 items on a 7-point scale ranging from 7, “I totally agree” to 1, “I totally disagree” (Cronbach alpha=.97) [30,45].

User experiences (also known as user perceptions) [30] were measured by four concepts: trustworthiness, enjoyment, active trust, and design aesthetic. *Trustworthiness* (eg, “I trust the information presented on this website”) was measured by 3 items on a 7-point scale ranging from 7, “I totally agree” to 1, “I totally disagree” (Cronbach alpha=.87). *Enjoyment* (eg, “I found my visit to this website enjoyable”) was measured by 3 items on a 7-point scale ranging from 7, “I totally agree” to 1, “I totally disagree” (Cronbach alpha=.92). *Active trust* (eg, “I would act on the information presented on this website if needed”) was measured by 3 items on a 7-point scale ranging from 7, “I totally agree” to 1,“I totally disagree” (Cronbach alpha=.91). *Design aesthetic* (eg, “The whole design of the website/program is attractive”) was measured by 3 items on a 7-point scale ranging from 7, “I totally agree” to 1, “I totally disagree” (Cronbach alpha=.93) [30].

Tailored-processing mechanisms were measured by five concepts. *Attention* for the tailored advice (eg, “The advice was interesting”) was measured by 4 items on a 7-point scale ranging from 7, “I totally agree” to 1, “I totally disagree” (Cronbach alpha=.89). *Comprehension* of the advice (eg, “The advice was clear to me”) was measured by 4 items on a 7-point scale ranging from 7, “I totally agree” to 1, “I totally disagree” (Cronbach alpha=.91). *Self-reference* towards the advice (eg, “The advice was personally relevant for me”) was measured by 4 items on a 7-point scale ranging from 7, “I totally agree” to 1, “I totally disagree” (Cronbach alpha=.91). *Appreciation* of the advice (eg, “I appreciated the advice”) was measured by 3 items on a 7-point scale ranging from 7, “I totally agree” to 1, “I totally disagree” (Cronbach alpha=.94). *Processing* of the advice (eg, “The advice encouraged me to think more about smoking cessation”) was measured by 4 items on a 7-point scale ranging from 7, “I totally agree” to 1, “I totally disagree” (Cronbach alpha=.93) [21].

An overall grade for the advice was measured by 1 item asking participants to give an overall score for the provided advice from 1 (very bad) to 10 (very good).

### Statistical Analysis

First, descriptive analyses were used to determine the sample’s characteristics. To test whether any baseline differences existed between the video and text condition, *t* tests were performed for interval scaled variables, whereas Chi-square tests were conducted for categorical variables. Additionally, the same analyses were executed to investigate whether baseline differences existed between higher educated and lower educated participants. Those variables that differed between condition and educational levels were included as covariates in all further analyses.

Second, two-way analyses of co-variance (ANCOVA) were carried out to assess whether any differences existed in message-processing mechanisms according to delivery strategy and educational level. The same analyses were executed to detect any differences in future adherence according to delivery strategy and educational level.

Last, a linear regression analysis was conducted to determine the unique predictive power of delivery strategy and educational level on future adherence when user experiences were included as independent predictors in the analysis. All analyses were conducted with SPSS 17.0.

In the main analyses, we used the sample of the 139 participants who stayed more than 5 minutes on the intervention website. These analyses were repeated using an intention-to-treat analysis of the data from all 204 participants to verify whether we did not introduce a selection bias by our restriction criterion.

## Results

### Sample Characteristics

There were no significant differences between participants in the video and text condition with regard to gender, educational level, age, cognitive processing, addiction level, and intention to quit smoking.

Participants in the video condition spent more time on the website in comparison with participants in the text condition (*t* (137) =5.06, *P*<.001), most probably due to the fact that the video condition lasted slightly longer than the text condition. Age of participants included in the analysis varied from 20 to 72 years (mean 47.39; SD 11.94). Overall, men were slightly underrepresented (37.4%). A description of the overall sample at baseline can be found in Table 1.

**Table 1:** Sample characteristics of adult Dutch smokers (N=139).

		Overall sample	Text condition	Video condition	*P*
**Gender, n (%)**					.42
	Female	87 (62.6 %)	44 (59.5%)	43 (66.2%)	
	Male	52 (37.4%)	30 (40.5%)	22 (33.8%)	
**Educational level, n (%)**					.45
	Low	68 (48.9%)	34 (45.9%)	34 (52.3%)	
	High	71 (51.1%)	40 (54.1%)	31 (47.7%)	
**Age, mean (SD)**		47.39 (11.94)	46.66 (11.90)	48.22 (12.03)	.45
**Cognitive processing, mean (SD)**		3.56 (0.61)	3.55 (0.62)	3.57 (0.60)	.83
**FTND score (1-10), mean (SD)**		3.57 (2.54)	3.41 (2.52)	3.75 (2.57)	.43
**Readiness to quit, n (%)**					.93
	Within 1 month	26 (18.8%)	14 (19.2%)	12 (18.5%)	
	Within 1-3 months	43 (31.25%)	22 (30.1%)	21 (32.3%)	
	Within 4-6 months	28 (20.3%)	14 (19.2%)	14 (21.5%)	
	Within 1 year	24 (17.4%)	12 (16.4%)	12 (18.5%)	
	Within 1-5 years	15 (10.9%)	10 (13.7%)	5 (33.3%)	
	After 5 years	2 (1.4%)	1 (1.4%)	1 (1.5%)	
**Time spent on website (min), mean (SD)**		8.11 (12.82%)	7.15 (9.82%)	7.81 (16.24%)	< .001

Table 2 shows that participants with either a high or low educational level did not differ with respect to condition, gender, readiness to quit smoking, and time spent on the website. However, educational level differed significantly with respect to age (*t* (137)=1.90; *P*=.05) and nicotine addiction (*t* (136)=4.69; *P*<.001). Lower educated participants were older and more addicted to nicotine compared to higher educated participants. Participants with a higher educational level scored significantly higher on cognitive processing than those with a lower educational level (*t* (137)=-5.00; *P*<.001).

**Table 2:** Differences of characteristics between lower and higher educational levels (N=139).

		Overall sample	High level	Low level	*P*
**Condition, n (%)**					.45
	Text	74 (53.2%)	40 (56.3%)	34 (50.0%)	
	Video	65 (46.8%)	31 (43.7%)	34 (50.0%)	
**Gender, n (%)**					.39
	Female	87 (62.6%)	42 (59.2%)	45 (66.2%)	
	Male	52 (37.4%)	29 (20.9%)	23 (33.8%)	
**Age, mean (SD)**		47.39 (11.94)	45.52 (13.63)	49.34 (9.60)	.05
**Cognitive processing, mean (SD)**		3.56 (0.61)	3.79 (0.56)	3.31 (0.57)	<.001
**FTND score (1-10), mean (SD)**		3.57 (2.54)	2.65 (2.45)	4.54 (2.27)	<.001
**Readiness to quit, n (%)**					.109
	Within 1 month	26 (18.8)	17 (23.9%)	9 (13.4%)	
	Within 1-3 months	43 (31.2%)	27 (38.0%)	16 (23.9%)	
	Within 4-6 months	28 (20.3%)	12 (16.9%)	16 (23.9%)	
	Within 1 year	24 (17.4%)	8 (11.3%)	16 (23.9%)	
	Within 1-5 years	15 (10.9%)	6 (8.5%)	9 (13.4%)	
	After 5 years	2 (1.4%)	1 (1.4%)	1 (1.5%)	
**Time spent on website, mean (SD)**		12.82 (8.11)	13.49 (9.92)	12.13 (5.62)	.33

### Differences in Message-Processing Mechanisms

To test possible interaction effects of delivery strategy and educational level on message-processing mechanisms, ANOVAs were conducted using each of the five message-processing measures as dependent variables. For the five measures, no interaction effects were found between delivery strategy and educational level. Subsequently, main effects of delivery strategy were tested on message-processing measures. As shown in Table 3, none of the measures approached significance. Furthermore, main effects of educational level were tested on message-processing measures. As shown in Table 4, lower educated participants devoted more attention to the tailored advice compared to higher educated participants (*F*(1,138)=3.97; *P*=.05). Also, the extent to which participants processed the information was shown to be higher among lower educated groups (*F*(1,138)=4.58; *P*=.04). No differences between lower and higher educated smokers could be found with regard to understanding, self-reference, appreciation, and the overall grade for the advice.

**Table 3:** Differences in variances of delivery strategy with regard to message-processing mechanisms (N=139).

Delivery strategy					
	Text group Mean (SD)	Video group Mean (SD)	F	*P*	η^2^
Attention	4.85 (1.43)	4.83 (1.28)	.00	.97	.000
Comprehension	5.50 (1.12)	5.41 (1.04)	1.15	.29	.008
Self-reference	4.93 (1.47)	4.97 (1.20)	.06	.81	.000
Appreciation	5.23 (1.43)	5.08 (1.32)	.40	.53	.003
Processing	4.56 (1.54)	4.83 (1.38)	.97	.33	.007
Grade advice	7.19 (1.36)	6.91 (1.56)	1.89	.17	.014

**Table 4:** Differences in variances of socioeconomic status with regard to message-processing mechanisms (N=139).

Educational Level					
	High level	Low level Mean (SD)	F	*P*	η^2^
Attention	4.51 (1.46)	5.21 (1.14)	3.97	.05	.029
Comprehension	5.41 (1.16)	5.54 (0.98)	.02	.67	.001
Self-reference	4.74 (1.42)	5.19 (1.23)	2.10	.15	.016
Appreciation	4.88 (1.50)	5.47 (1.16)	1.75	.19	.013
Processing	4.32 (1.53)	5.09 (1.31)	4.58	.04	.033
Grade advice	6.92 (1.46)	7.21 (1.54)	1.45	.23	.011

### Differences in Future Adherence

To test for possible interaction effects of delivery strategy and educational level on future adherence, ANOVAs were conducted using each of the two measures as dependent variables. For both measures, interaction effects between delivery strategy and educational level were found to be insignificant. Next, main effects of delivery strategy were tested on future adherence. As shown in Table 5, the two measures did not approach significance. Furthermore, main effects of educational level on future adherence were conducted. As shown in Table 6, lower educated participants had a higher intention to visit the website again compared to participants with a higher educational level (*F*(1,138)=4.43; *P*=.04). Recommending the website to others did not differ among lower and higher educated smokers.

**Table 5:** Differences in variances of delivery strategy with regard to future adherence (N=139).

Delivery strategy					
	Text group Mean (SD)	Video group Mean (SD)	F	*P*	η^2^
Intention	4.61 (1.58)	4.54 (1.61)	.00	.95	.000
Recommendation	4.65 (1.63)	4.74 (1.58)	.03	.87	.000

**Table 6:** Differences in variances of socioeconomic status with regard to future adherence (N=139).

Educational level					
	High level Mean (SD)	Low level Mean (SD)	F	*P*	η^2^
Intention	4.23 (1.76)	4.96 (1.28)	4.43	.04	.032
Recommendation	4.37 (1.72)	5.05 (1.41)	2.72	.10	.020

### Predictors of Future Adherence

In order to test whether delivery strategy, educational level, age, smoking dependency, cognitive processing, and time spent on the website were independent predictors of future adherence, multiple regression analysis was executed. User experiences were included as well as independent predictors in the analysis to determine the unique predictive power of delivery strategy and educational level in addition to user experiences. Therefore, the first model consisted of user experiences. Second, we investigated whether these results would change after controlling for delivery strategy, educational level, age, gender, and smoking behavior. Results of the multiple regression analysis are presented in Table 7. User experiences did not alter the observed results.

**Table 7:** Multiple regression analysis for future adherence (N=139).

Predictor variable (Beta)	Intention	Recommendation
Trustworthiness	-.145	-.093
Enjoyment	-.057	-.171
Active trust	-.544^b^	-.487^b^
Design aesthetics	-.128	-.158^b^
Condition	-.053	-.010
SES	-.156	-.032
Age	-.086	.017
Gender	-.061	-.028
Smoking dependency	-.008	-.015
Cognitive processing	.015	-.033
Time on the website	.006	.075
R square	.697	.721

^a^ *P*<.01.

^b^ *P*<.05.

### Intention-to-Treat Analysis

The intention-to-treat analysis revealed no different results with regard to sample characteristics. For both, message-processing mechanisms and future adherence interaction effects between delivery strategy and educational level were found to be insignificant. Next, main effects of delivery strategy and educational level were tested on message-processing mechanisms and future adherence. We found a significant difference between the two message methods only regarding the concept of comprehension. Participants in the text condition showed a higher comprehension of the tailored messages compared to people in the video condition (*F*(1,201)=4.34; *P*=.04). However, the results of this analysis should be carefully interpreted since people staying less than 5 minutes on the website could not possibly have read the advice in-depth.

## Discussion

The first objective of the present study was to investigate whether differences exist in message-processing mechanisms according to delivery strategy and education level for a computer-tailored smoking cessation intervention. Results revealed that delivery strategy did not interact with education on message-processing mechanisms. This means that delivery strategy had no influence on the processing of the message by participants with different educational levels. Moreover, the processing of the tailored information did not differ between the two conditions. The idea that the processing of computer-tailored information would depend on the delivery strategy did not hold for this computer-tailored intervention. This conclusion is in line with results found in a recent study concerning physical activity, which demonstrated no differences between video- and text-generated computer-tailored messages [46]. One explanation for these findings may be the exposure time of the intervention. Participants may have needed to follow more than one session of the intervention in order to detect differences between the two delivery modes. Next, our findings revealed that lower educated smokers paid more attention to the tailored advice and seemed to process information more deeply. As such, lower educated smokers seemed to be more involved in the computer-tailored intervention than higher educated smokers. The findings imply that the current intervention session succeeded in approaching those smokers in the general population who might profit the most from these computer-tailored interventions. Our findings correspond with those from a recent study that found that lower educated participants were more likely to finish a module of a computer-tailored lifestyle intervention [47]. We did not find other differences between educational groups with respect to the other message-processing mechanisms: comprehension, self-reference, and appreciation, which is contrary to previous research that did find higher appreciation rates of computer-tailored advice among lower educated participants [48].

The second objective of this study was to examine whether differences exist in future adherence (intention to revisit, recommend the website to others) with regard to delivery strategy and education. The results revealed no interaction between delivery strategy and education on the intention to adhere; implying that a different delivery strategy did not influence future adherence of lower and higher educated smokers. Again, delivery strategy was found to have no particular effect on future adherence. However, the results demonstrated that lower educated smokers were more inclined to revisit the website compared to higher educated smokers. Although not significant, our findings revealed a slightly better appreciation of the website by lower educated participants, which may have contributed to the positive intention to revisit the website. As already mentioned, this is in line with findings of a previous study that lower educated participants were more inclined to initiate a lifestyle program [47]. Additionally, our lower educated participants were slightly less motivated (*P*<.11) to quit immediately, which also could explain a need for continued help to prepare them to quit in the future. Although lower educated smokers were more inclined to visit the website again, we could not find any educational differences with regard to recommending the website to others. As the results indicate that the “word of mouth”’ strategy might be not sufficient to recruit participants for Internet interventions, other recruitment strategies may also be needed such as recruitment through general practitioners [49]. A question raised by our results is why delivery strategy did not influence message-processing mechanisms and future adherence among different educational groups. One explanation could be that we recruited persons via an Internet research agency, which may have more innovative members that have already an open mind to Internet interventions. It might be possible that for those people, delivery mode did not differ that much. It is conceivable that delivery mode preference might have been different if we had recruited participants via other strategies, eg, newspaper advertisements. Furthermore, it might be possible that participants were not engaged by the design of the intervention components. The two conditions might have been presented in a more engaging way with further images, graphics, hyperlinks, and other animation effects. However, an important precondition for our experimental design was that the information of the video condition was contingent on the information provided by text condition to reveal the added effect of presenting information through a video format.

Our findings reveal that the tailored advice given was more positively evaluated by lower educated smokers. This outcome is in contrast with earlier studies indicating that web-based programs may result in a digital divide between lower and higher educated groups. Perhaps high Internet use among the Dutch (over 90%) may explain these effects [37]. Moreover, since our less educated smokers often show higher addiction rates, fewer quit attempts, and more relapses [14,15], approaching them via Internet may have added potential.

### Study Limitations

The present study is subject to certain limitations. First, as with many health communication studies, we could not objectively assess quality of information processing as we could not measure it. Yet we did assess how long participants stayed on the website via server registrations and therefore could exclude all participants who briefly visited the intervention website and probably did not accurately process the given information. Second, our sample size was limited. Additional longitudinal research with a larger sample size is needed to investigate the role of delivery strategy in tailored information and to examine whether smokers will actually revisit the website and whether this will result in the ultimate goal of behavior change.

### Conclusions

Delivery strategy did not play a role in the processing of the tailored information. Lower educated participants showed higher attention and processing levels. Lower educated participants were also more inclined to visit the intervention website again compared to higher educated participants. Due to the fact that all participants were members of an Internet research panel, the results can be applied only to people who are already regularly using the Internet. This study can be seen as an important first step to assess the influence of delivery strategy among different educational groups and especially among lower educated smokers in the Netherlands. Effective smoking cessation interventions are important to decreasing the gap between lower and higher educated smokers. Yet, longitudinal studies with larger sample sizes are needed to see whether these counterintuitive findings still hold true and to further assess additional aspects that we could not assess, such as actual revisit and long-term behavioral effects of the two strategies among lower and higher educated smokers in order to improve computer-tailored smoking cessation interventions.
